# Genome Editing of the *NF-YA8* Gene Modifies Tomato Plant Architecture and Fruit Traits

**DOI:** 10.3390/plants14121826

**Published:** 2025-06-13

**Authors:** Nestor Petrou, Nikoleta Tsigarida, Zoe Hilioti

**Affiliations:** Institute of Applied Biosciences, Centre for Research and Technology Hellas, GR 57001 Thermi, Greece; nestorpetrou@certh.gr (N.P.); ntsigarida@certh.gr (N.T.)

**Keywords:** genome editing, ZFN, NF-YA8, seedling, plant architecture, tomato, fruit, breeding

## Abstract

Genome editing has revolutionized plant science, providing an unprecedented ability to precisely manipulate plant genomes. For this study, genome editing was utilized to target and modify the NF-YA8 transcription factor (TF) in tomato plants (*Solanum lycopersicum* L. var. Heinz 1706). The primary objective of this research was to introduce targeted mutations in a non-transgenic manner to the *NF-YA8* gene, which encodes the alpha subunit of the Nuclear Factor-Y (NF-Y) heterotrimeric TF, and explore its potential for developing new and improved tomato varieties. Through the transient expression of custom-engineered zinc finger nucleases (ZFNs) in tomato seeds, mutations were successfully introduced in the target gene. The recovered mutant *NF-YA8* coding sequences showed a significant level of similarity to the wild type, with a range of 86.9% to 98.21%. Genotyping M2 lines revealed monogenic mutations at or near the intended target site. Phenotypic changes were also evident in both vegetative and reproductive stages of plants. The research revealed that NF-YA8 functions as a high-level regulator, orchestrating a developmental cascade that influences key agronomic traits throughout the plant’s life cycle, including cotyledon development, stem architecture, inflorescence architecture, flowering time, and fruit size and shape.

## 1. Introduction

The development of genome editing (GE) technologies has made it possible to precisely alter genomes and investigate gene function. ZFNs were originally invented by Chandrasegaran’s group [1], and their utilization was pioneered by Dana Carroll and his team [2]. This breakthrough approach paved the way for targeted gene editing, completely transforming the realm of genetic engineering and impacting various fields such as medicine, agriculture, and biotechnology. ZFN-based genome editing technologies have been shown to be efficient and precise in a wide range of plant species, including tobacco, *Arabidopsis*, maize, and soybean [3] ZFNs utilized the DNA-binding component of zinc finger proteins fused with the cleavage component of the FokI restriction enzyme. Previously, the use of ZFN technology in tomato (*S. lycopersicum*) seeds targeted the disruption of the *L1L4*/*NF-YB6* gene, a crucial component of the NF-Y TF complex [4]. The disruption of *L1L4* in tomato had a significant impact on both the vegetative and reproductive structures of the plant [4]. The resulting mutant lines (M4) displayed a variety of modifications in their fruits, including changes in seed storage proteins, fruit shape, increased levels of fructose, and decreased levels of oxalic acid, a known antinutrient [5]. In *Arabidopsis,* LEC1/NF-YB9, L1L/NF-YB6, and bZIP67 work together to regulate the expression of genes with ABA-responsive elements (ABREs) in their promoter regions during the development of embryos [6]. NF-Y proteins, which exist as heterotrimers, are pivotal TFs that have been conserved through evolution and play a critical role in regulating gene expression by binding to the CCAAT box located in gene promoters [7,8]. These complexes comprise the subunits NF-YA, NF-YB, and NF-YC. The NF-YB and NF-YC subunits form a heterodimer in the cytoplasm, which then translocate to the nucleus where they recruit the NF-YA subunit to form the mature complex [9,10]. Once formed, the NF-YA subunit (also known as HAP2 or CBF-B) confers precise sequence specificity, enabling it to specifically bind to the CCAAT box on target gene promoters [11]. It is estimated that approximately 30% of eukaryotic promoters contain CCAAT boxes [12,13].

While in yeast and animals, each subunit of NF-Y is encoded by a single gene, in plants, the subunits of NF-Y are encoded by gene families [7,8,14]. The tomato *NF-YA* gene family consists of 10 members encoding proteins of varying lengths (ranging from 154 to 325 amino acids) [15]. The large *NF-YA* gene family and the different expression patterns in tissues and developmental stages pose a challenge in determining the specific role of each *NF-YA* gene in the tomato plant. The application of virus-induced gene silencing (VIGS) in tomato has demonstrated that the ripening process is influenced by five *NF-Y* genes, specifically two members from the NF-YB subgroup (*Solyc06g069310*, *Solyc07g065500*) and three members from the *NF-YA* subgroup (*Solyc01g087240*, *Solyc08g062210*, *Solyc11g065700*) [5]. It is intriguing that the NF-YA subgroup, which has an impact on tomato fruit ripening, did not show increased expression in fruits [15,16]. This finding prompts inquiries about the intricate regulation of NF-Y genes and their potential role in other developmental stages. It underscores the importance of additional investigations to comprehensively grasp the mechanisms and roles of this gene family in tomato plants.

The aim of this study was to understand the role of NF-YA8 (*Solyc08g062210*) and its potential in breeding improved tomato varieties. Using ZFN-mediated genome editing, we created non-transgenic mutations in the DNA-binding domain of NF-YA8 in a high-throughput manner. NF-Y is a highly conserved protein complex in eukaryotes that, while composed of three subunits (A, B, and C), is thought to rely primarily on its A subunit for promoter recognition. Analysis of the resulting M1 and M2 generations derived from these mutations revealed that NF-YA8 significantly impacts key agronomic traits related to both vegetative and reproductive growth, underscoring its potential as a target for developing improved tomato varieties.

## 2. Results

### 2.1. High-Frequency ZFN-Induced Mutagenesis in S. lycopersicum Seeds

**Figure 1:**
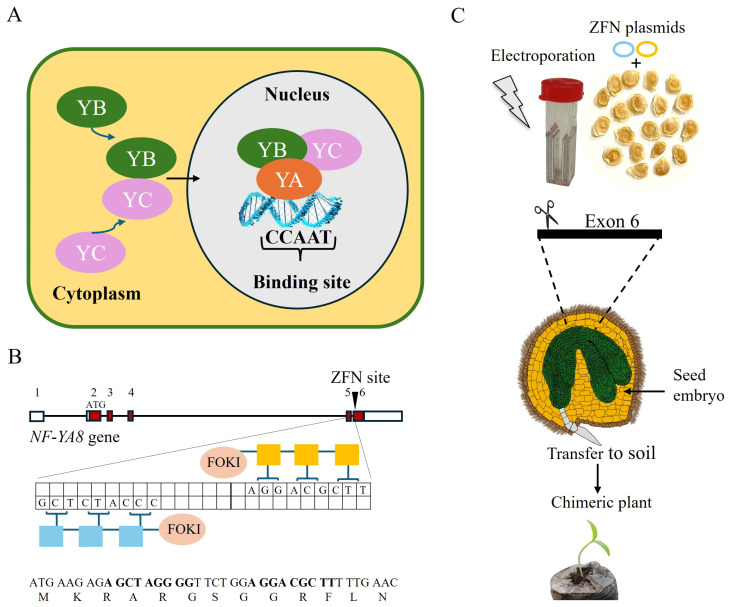
ZFN-mediated genome editing of the *NF-YA8* gene in *S. lycopersicum* seeds. (**A**) The target gene, *NF-YA8*, encodes the A subunit of the heterotrimeric NF-Y transcription factor. (**B**) A schematic illustrates the design of zinc finger nucleases (ZFNs) targeting *NF-YA8*. The ZFN target site is in exon 6, with the left ZFN binding the bottom strand and the right ZFN binding the top strand. The translated targeted sequence, including the ZFN recognition sites (bolded), is included below. (**C**) Germinating seeds were electroporated with ZFNs and subsequently planted in soil.

*NF-YA8*, located on chromosome 8, encodes the A subunit of the heterotrimeric NF-Y transcription factor (Figure 1A).

The intron–exon structure shows that this gene contains six exons, with the start codon (ATG) found in exon 2 (Figure 1B), which is the regulatory exon. All predicted *NF-YA8* isoforms, according to Ensembl Plants, share exon 6, making it a suitable region for modification. The target site was specifically chosen at the start of this common exon. To target exon 6, a right zinc finger nuclease (ZFN) was engineered to bind the sequence 5′-AGGACGCTT-3′ on the top strand, and a left ZFN was designed to bind the sequence 5′-CCCATCTCG-3′ on the bottom strand (Figure 1B). To detect mutations at the *NF-YA8* locus in genomic DNA and cDNA of M1 generation plants, and to evaluate potential off-target effects, we used PCR-based techniques coupled with high-resolution melting (HRM) analysis for initial screening. In most cases, gel electrophoresis showed that DNA amplicons were of similar sizes across different samples. This screening method was crucial due to the lack of a selection method directly linked to the transient expression of the ZFNs. The same screening process was repeated on the fertile M2 generation of the *nf-ya8* M1s.

**Figure 2:**
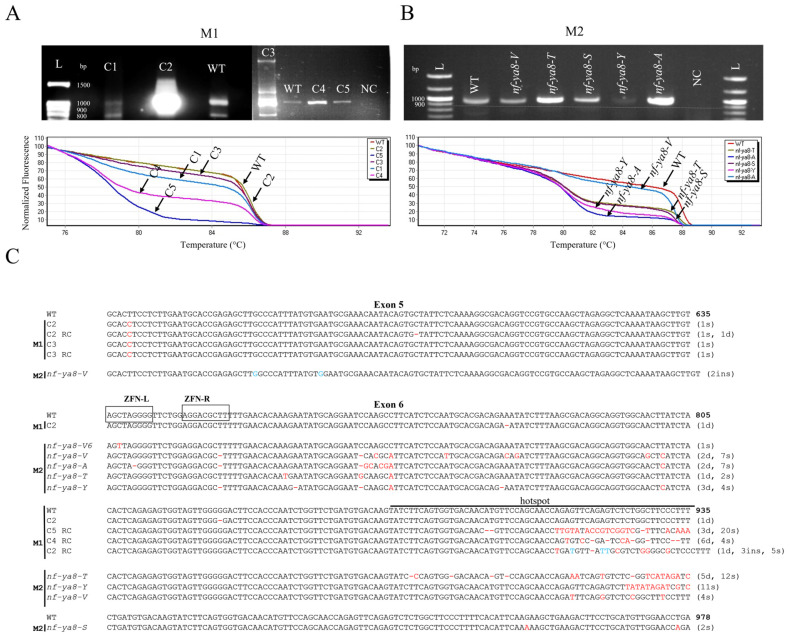
Mutation screening of the target gene *NF-YA8*. (**A**) DNA from chimeric founder plants (M1 generation; C1–C5) was screened for mutations using PCR amplification followed by agarose gel electrophoresis. Wild-type DNA (WT) and a 100 bp ladder (L) served as controls. NC: negative control. High-resolution melting (HRM) analysis further identified *NF-YA8* mutations, as evidenced by shifted DNA melting curves in M1 plants relative to the wild-type control (red curve; lower panel). (**B**) The same PCR/electrophoresis and HRM analysis was performed on DNA from M2 plants (*nf-ya8-V*, *-T*, *-S*, *-Y*, -A). Again, WT DNA and a 100 bp ladder (L) were used as controls, and the lower panel shows shifted melting curves in M2 plants compared to the wild-type control (red curve). (**C**) A multiple sequence alignment compared the *NF-YA8* variants identified in the M1 and M2 generations, resulting from ZFN-induced mutations. The ZFN target sites are indicated by boxes. The following abbreviations denote mutations: "s" for substitution, "d" for deletion, and "ins" for insertion. Insertions are highlighted in blue, deletions are represented by red dashes, and substitutions are shown with red letters.

High-resolution melting (HRM) analysis successfully identified mutations at the *NF-YA8* gene locus in all chimeric M1 plants (Figure 2A). Screening of the M2 progeny using the same protocol yielded amplicons of consistent length (small deletions, mostly substitutions) based on gel electrophoresis (Figure 2B). However, high-resolution melting (HRM) analysis demonstrated improved separation of the DNA melting curves between different samples (Figure 2B, bottom panel), indicating natural selection.

For sequencing purposes, PCR products from M1 (pooled sample) and M2 plants were cloned. Sanger sequencing was performed on amplicons exhibiting significant differences from the wild-type sequence in high-resolution melting (HRM) analysis. This revealed insertions, deletions, and single-nucleotide substitutions primarily located in exon 6 (positions 692–935), with some also present in exon 5 (Figure 2C). The distribution of mutations varied across different mutant lines, with *nf-ya8*-*V* and *nf-ya8*-*T2* showing a higher frequency of mutations compared to others. The analysis of over 90 sequences revealed a variety of mutations, encompassing single-nucleotide variants (SNVs), insertions/deletions (indels), and complex mutations. SNVs were the most frequent (51.5%), followed by indels, which comprised a substantial 39.4% of all mutations. A notable proportion of observed indels (24.2%) were predicted to induce frameshift mutations and loss-of-function mutations (6.1%). The remaining mutations were categorized as complex. Within the ZFN target site of exon 6, we identified a C-to-T transition at position 692, a guanine deletion at position 695, and a thymine deletion at position 712. Notably, a mutation hotspot with multiple indel mutations was found between positions 873 and 935. Interestingly, the variety of mutations within this hotspot region was reduced in the M2 generation.

Two out of seventy ZFN-treated plants (2.85%) displayed a poor growth phenotype, characterized by tiny cotyledons and delayed development. These two plants were selected for molecular analysis. As anticipated, the sequencing of exon 6 in these plants revealed mutations, primarily small insertions and deletions (indels) indicative of NHEJ-mediated repair. Unexpectedly, sequence analysis of exon 2 in the same two plants also revealed mutations, including single-nucleotide substitutions, insertions, and deletions. Specifically, plant sample C2 RC showed a single-nucleotide substitution (C to T at the beginning of the sequence) and two single-nucleotide deletions following the sequences “CTACT” and “TG”. Plant sample C9 exhibited a more complex mutation pattern, including a substitution (G to A), a deletion (A after “CAA”), and an insertion (GGG after “ATCATT”). Both C2 RC and C9 RC harbored mutations within exon 2 that are likely to have significant consequences for protein function. The frameshift mutations, caused by the deletions and compound insertion/deletions, are unlikely to maintain any functional similarity to the wild-type protein. Notably, in silico analysis did not identify any potential ZFN cleavage sites within exon 2. The co-occurrence of mutations in both exon 2 and exon 6 within the same two phenotypically aberrant plants strongly suggests a linked mutational event. We propose that the formation of a chromatin loop brings these two distant DNA regions into close proximity, allowing the ZFN to cleave both sites simultaneously or in close succession. The significance of thoroughly analyzing the entire gene sequence is emphasized by this study to fully understand the potential consequences of the identified mutations.

Overall, at the DNA level, the recovered mutant *NF-YA8* coding sequences showed a significant level of similarity to the wild type, with a range of 86.9% to 98.21%.

**Figure 3:**
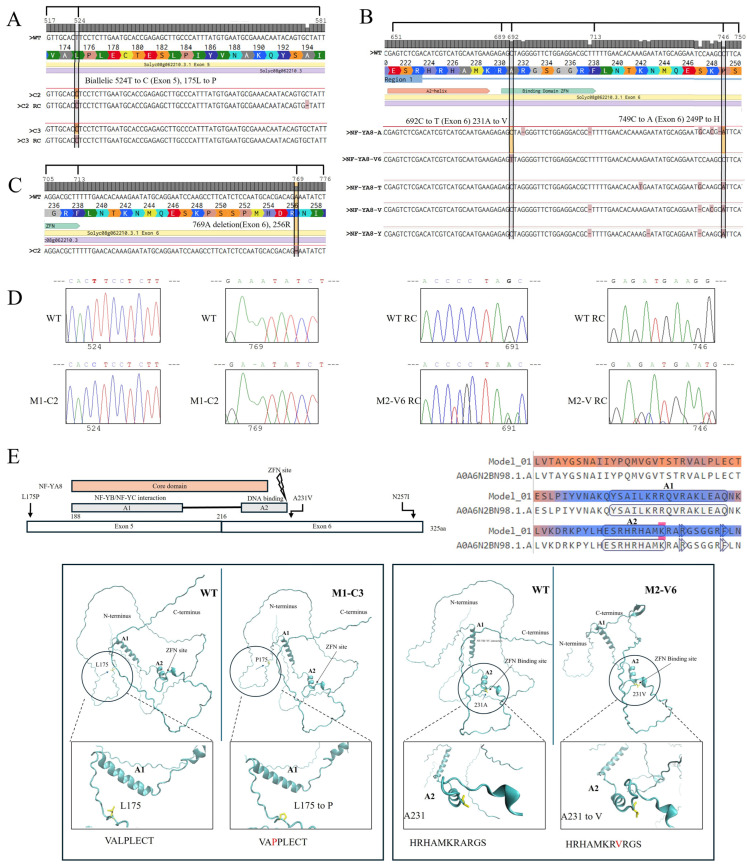
Sequences of selected ZFN-induced mutations in M1 and M2 plants. (**A**) The C2 and C3 samples exhibited a biallelic mutation at position 524 (T to C) in exon 5 situated 165 nucleotides upstream of the binding site of the coding sequence, resulting in an amino acid substitution, 175L to P. Additionally, a C deletion at position 581 in exon 5 was identified in C2 RC. (**B**) In exon 6, a C-to-T transition at position 692 was observed, leading to an amino acid change, 231A to V. Notably, the 692C-to-T mutation occurred within the ZFN target site (689–713). (**C**) The C2 sample exhibited a deletion of adenine at position 769 in exon 6, which is 56 nucleotides downstream of the ZFN site, leading to the amino acid modification 256R. These sequence modifications, occurring within and near the genome editing target region, may have functional implications for *NF-YA8* gene regulation and tomato development. Symbols and abbreviations: RC, reverse complement; S, sample; P, primer. (**D**) Chromatograms showing the detected mutations. (**E**) Overview of the locations of the mutations within the protein’s conserved domains, including the well-conserved core domain with predicted A1 and A2 helices, the NF-YB/NF-YC interaction domain, the DNA binding region, and the ZFN target site. A SWISS-MODEL alignment of the NF-YA8 amino acid sequence (Model_01) with its template shows the predicted A1 and A2 helices enclosed in rectangles. Τhe predicted structure from AlphaFold was used to compare the original (WT) and mutated forms from M1 (M1-C3) and M2 (Vigorous) generations. The analysis focused on structural differences near the A1 and A2 helices. The native structure features L175 and A231, while the mutant clone C3 contains P175 and the Vigorous A231. Insets (squares) provide close-up views highlighting these residue-specific changes (circles).

Detailed analysis of selected tomato *NF-YA8* gene mutations from the M1 and M2 generations and their predicted effects on the resulting protein is presented (Figure 3). In samples C2 and C3, we identified a biallelic T-to-C mutation at position 524 in exon 5, leading to a leucine-to-proline substitution at amino acid 175 (175L to P). Furthermore, sample C2 RC exhibited a cytosine deletion at position 581, also in exon 5. Within exon 6, at position 692, a C-to-T transition resulted in an alanine-to-valine substitution at amino acid 231 (231A to V); this mutation lies within the ZFN target site (positions 689–713). Sample C2 showed an adenine deletion at position 769 in exon 6, causing a modification at amino acid 256 (256R), located 56 nucleotides downstream of the ZFN binding site. These sequence alterations, occurring within or near the genome editing target region, suggest potential functional consequences for *NF-YA8* gene regulation and tomato development. As illustrated in Figure 3E, which maps the mutations onto the protein’s domains (including the NF-YB/NF-YC interaction domain, the DNA binding region, and the ZFN target site), the well-conserved core domain with predicted A1 and A2 helices remained unaffected by any of the observed mutations. The A1 domain is crucial for NF-Y protein complex formation, enabling the subunits NF-YB and NF-YC to bind together. The A2 domain determines the protein’s specific DNA binding, allowing it to recognize and bind to CCAAT box sequences. Homology modeling using SWISS-MODEL allowed for comparative amino acid-level analysis of NF-YA8 protein mutants generated by ZFNs. The L175P mutation in NF-YA8 (M1-C3), which changes the VALPLECT motif to VAPPLECT, is predicted to significantly impact NF-Y complex structure, stability, and function (Figure 3E). Specifically, the introduction of proline at this position may disrupt proper folding of the A1 helix and weaken the interaction between NF-YA and the NF-YB/YC heterodimer, potentially leading to reduced DNA binding and decreased NF-Y target gene expression. For the Vigorous mutant (M2), a model was built using the AlphaFold-derived structure of *Capsicum annuum* var. *glabriusculum* (accession A0A5P9S0S9_CAPAN) due to its higher sequence homology to M2 than the tomato NF-YA8 protein. Visualization using VMD further explored structural differences between these models. Although the sequence change in Vigorous mutant HRHAMKRARGS to HRHAMKRVRGS is conservative, the A231V mutation at the end of the A2 helix within the DNA-binding domain (DBD) may also compromise NF-YA structure, stability, and DNA binding affinity. The addition of the bulkier valine side chain at position 231 could disrupt local packing, alter hydrogen bonding, and destabilize the A2 helix, a region critical for proper DNA interaction. Even subtle structural alterations in this region can diminish NF-YA’s affinity and specificity for the CCAAT box, ultimately leading to altered gene expression.

The M2 generation exhibited a wide spectrum of genetic variations and largely maintained a heterozygous state. This was due to the intricate segregation of numerous alleles and the diverse distribution of the introduced genetic modifications during inheritance. Our bioinformatic analysis did not identify any likely off-target cleavage sites (Text S1).

Furthermore, we screened potential off-target regions (*Solyc01g006930.2.1*, *Solyc01g087240.2.1*, and *Solyc11g065700.1.1*) in M1 plants and their progeny, focusing on sites with 11–44% variation compared to the intended target site. This screening revealed no evidence of off-target mutations.

### 2.2. The Regulatory Role of NF-YA8 in Seedling Growth and Development

**Figure 4:**
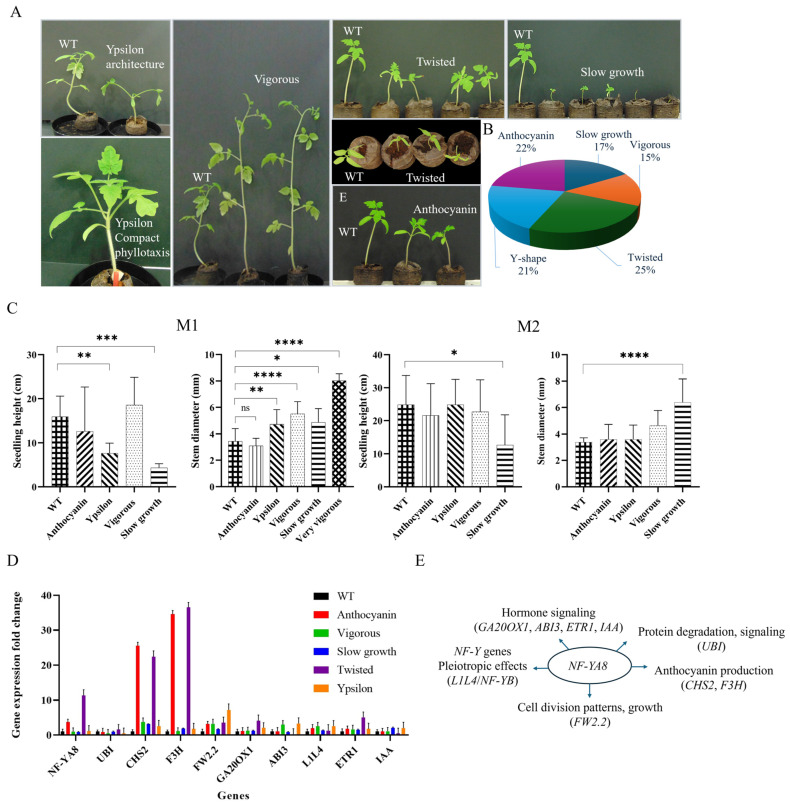
Phenotypic categories and growth characteristics in *NF-YA8* gene disruption mutants. (**A**) Plants exhibiting ypsilon architecture and the wild type (top); a variation of ypsilon architecture characterized by a compact phyllotaxis (bottom); the vigorous growth category compared to the wild type; plants with twisted cotyledons, shown both in comparison to the wild type (top) and from a top-down perspective (bottom); the slow growth category and the rich-in-anthocyanin category. (**B**) The pie chart illustrates the distribution of the five *nf-ya8* mutant categories. (**C**) Seedling height (cm) and diameter were measured in both the M1 (*n*= 7–17) and M2 (*n* = 6) generations for each *nf-ya8* mutant group. Data are presented as mean ± SD, with statistical significance determined by one-way ANOVA. The differences among the groups were determined using Tukey’s post-hoc test for multiple comparisons. Significance levels are indicated by asterisks as follows: * *p* < 0.05, ** *p* < 0.001, *** *p* < 0.0001, and **** *p* < 0.0001. (**D**) Quantitative PCR with EvaGreen dye was used to analyze the expression of specific tomato genes related to hormone signaling (*GA20OX1*, *ABI3*, *ETR1*, *IAA*), anthocyanin production (*CHS2*, *F3H*), development (*L1L4*), protein degradation (*UBI*), and cell division (*FW2.2*) in 7-day-old wild-type (WT) and *nf-ya8* mutant (M2 generation) seedlings. Gene expression was normalized to the reference gene *EF1-α,* and fold change relative to the WT control (set to 1) was calculated using the 2^−ΔΔCt^ method. The graph displays the fold change in expression for each gene, with error bars representing the means ± SD of three biological replicates. (**E**) A model for the *NF-YA8* gene effects in tomato seedlings. The disruption of the TF leads to pleiotropic phenotypic variations mediated by the dysregulation of key developmental genes.

The creation of tomato (Heinz 1706) mutants using ZFNs resulted in a diverse array of seedling phenotypes in both the M1 and M2 generations. The initial generation (M1) resulting from the ZFN treatment displayed a diverse range of altered physical characteristics, allowing for categorization based on observed phenotypes (Figure 4A).

These categories included plants exhibiting elevated stem anthocyanin levels (*nf-ya8-A*), characterized by a distinct reddish-purple coloration; individuals with vigorous growth (*nf-ya8-V*), showing increased size and development; those with twisted cotyledons (*nf-ya8-T*), marked by abnormal leaf structure; plants exhibiting slow growth (*nf-ya8-S*), displaying reduced development rate; and finally, individuals with ypsilon-shaped stems (*nf-ya8-Y*), characterized by a unique stem bifurcation. The distribution of these phenotypes within the ZFN-treated population varied, with the twisted cotyledon phenotype (*nf-ya8-T*) being the most prevalent at 25%, followed by elevated stem anthocyanin (*nf-ya8-A*) at 22%, ypsilon-shaped stems (*nf-ya8-Y*) at 21%, and slow growth (*nf-ya8-S*) at 17% (Figure 4B). This phenotypic diversity suggests successful alteration of the target gene and highlights the varied impact of the ZFN-mediated modification on plant morphology. Subsequent analysis of the M2 generation revealed that some phenotypes, like *nf-ya8-V* and *nf-ya8-S*, were heritable. Furthermore, molecular analysis confirmed the presence of distinct mutations within the *NF-YA8* gene in these M2 mutants, ranging from nucleotide substitutions to insertions and deletions, especially within exon 6. These mutations often resulted in frameshifts and premature stop codons, likely leading to a complete loss of gene function in severely affected lines.

Finally, variations in growth patterns, specifically height and stem thickness, were observed in both M1 and M2 generations, with some mutants exhibiting exceptionally thick stems, further illustrating the regulatory role of NF-YA8 in plant growth and development. In the M1 generation, we measured the height and stem thickness of these mutants and compared them to wild-type seedlings. In addition, the *nf-ya8* mutants exhibited altered growth patterns, with variations observed in both height and stem diameter (Figure 4C). Notably, all *nf-ya8* mutant categories, except for *nf-ya8-A*, showed significantly thicker stems compared to the wild type. We identified a subset of *nf-ya8-V* mutants, designated "Extremely Vigorous" (*nf-ya8-V+*), displaying remarkably thick stems (8 mm in diameter) that were significantly larger than those of the wild type (3.5 mm). Unfortunately, these *nf-ya8-V+* mutants were sterile, precluding further analysis. In the subsequent M2 generation, *nf-ya8-S* mutants continued to display significant variation in both height and stem diameter relative to age-matched wild-type plants (Figure 4C). Therefore, our findings demonstrate that NF-YA8 is a crucial regulator of plant growth and development, and disruption of its function leads to significant phenotypic consequences.

To further understand the phenotypic differences between wild-type and *nf-ya8* mutant tomato seedlings, we analyzed the expression levels of several key developmental genes, such as *NF-YA8* (target gene), *CHALCONE SYNTHASE2* (*CHS2*), *FLAVANONE 3-HYDROXYLASE* (*F3H*), *FRUIT WEIGHT* (*FW2.2*), *GIBBERELLIN 20-OXIDASE 1* (*GA20OX1*), *ABSCISIC ACID INSENSITIVE 3* (*ABI3*), *LEAFY-COTYLEDON1-LIKE4* (*L1L4*), *UBIQUITIN* (*UBI*), *INDOLE-3-ACETIC ACID INDUCIBLE 2* (*IAA2*), and *ELONGATION FACTOR 1-α* (*EF1-α,* reference gene) (Figure 4D). The two-way ANOVA results for fold change in gene expression relative to the wild type (expression level = 1) indicated significant main effects of both genotype (variants) (*p* < 0.0001) and selected genes (*p* < 0.0001), as well as a significant interaction effect between genotype and gene (*p* < 0.001), suggesting that the *nf-ya8* variant effect on gene expression is gene-dependent. The biological relevance of these gene expression changes depends on the function of each individual gene in seedling physiology. For *NF-YA8* (target gene) itself, expression was considerably higher in the Anthocyanin (3.70) and Twisted (11.39) mutants. Conversely, lower *NF-YA8* expression was detected in the Vigorous (0.93), Slow growth (0.84), and Ypsilon (1.11) mutants. Expression patterns for *CHS2* and *F3H*, both genes involved in anthocyanin biosynthesis, were similar. The Anthocyanin phenotype showed the highest expression for both *CHS2* (25.6) and *F3H* (34.6). Expression levels in the other mutants, relative to the wild type, were as follows: For *CHS2*, Vigorous (3.78), Slow growth (3.14), Twisted (22.43), and Ypsilon (2.56); for *F3H*, Vigorous (1.1), Slow growth (1.85), Twisted (36.54), and Ypsilon (1.76). *FW2.2*, a gene related to fruit weight regulation, exhibited variable expression across the phenotypes. Expression was highest in the Ypsilon mutants (7.23) and, relative to the wild type, was 3.14 in Anthocyanin, 3.23 in Vigorous, 1.67 in Slow growth, and 3.56 in Twisted mutants. Similarly, *GA20OX1*, involved in gibberellin biosynthesis, showed differing expression levels across the mutants, with the highest expression in the Twisted mutants (4.12). Expression levels relative to the wild type were 1.11 in Anthocyanin, 1.2 in Vigorous, 1.23 in Slow growth, and 2.12 in Ypsilon mutants. *ABI3*, a gene involved in abscisic acid signaling, displayed the lowest expression in the Ypsilon mutants (0.24). Compared to the wild type, *ABI3* expression was 1.09 in Anthocyanin, 3.03 in Vigorous, 0.88 in Slow growth, and 0.46 in Twisted mutants. The gene *L1L4* (*NF-YB*), involved in growth and development, was most highly expressed in the Vigorous mutants (2.54). Expression levels relative to the wild type were 1.95 in Anthocyanin, 1.3 in Slow growth, 1.28 in Twisted, and 2.58 in Ypsilon mutants. The gene *ETR1*, involved in ethylene signaling, showed the highest expression in the Twisted mutants (5.07). Relative to the wild type, expression levels were 1.79 in Anthocyanin, 1.65 in Vigorous, 1.56 in Slow growth, and 1.74 in Ypsilon mutants. Expression of IAA, involved in auxin signaling, was lowest in the Twisted mutants (0.46). In contrast, expression levels, relative to the wild type, were 1.05 in Anthocyanin, 1.08 in Vigorous, 2.12 in Slow growth, and 1.99 in Ypsilon mutants. Finally, the expression levels of *UBI* (ubiquitin), a protein involved in protein degradation and signaling, were analyzed in several mutants. The Slow growth and Anthocyanin mutants displayed *UBI* expression levels similar to those observed in the wild type, suggesting that the genetic mutations affecting these phenotypes do not significantly impact *UBI* regulation. However, the Twisted mutant exhibited a noticeable increase in *UBI* expression (1.57), indicating a potential link between the mutated *NF-YA8* gene and the upregulation of *UBI*. Conversely, both Ypsilon and Vigorous mutants showed a marked decrease in UBI expression compared to the wild type, suggesting that the genes disrupted in these lines may play a role in positively regulating UBI expression. Overall, disruption of the *NF-YA8* gene in tomato seedlings leads to significant changes in gene expression, resulting in various phenotypic changes (Figure 4E). Specifically, NF-YA8 appears to repress anthocyanin production (as seen by upregulation of *CHS2* and *F3H*), regulates cell division patterns (indicated by altered *FW2.2* expression), and influences hormone signaling pathways (shown by the variable expression of *GA20OX1*, *ABI3*, *ETR1*, and *IAA*). NF-YA8 also impacts plant architecture through pleiotropic genes like *L1L4*. These findings suggest NF-YA8 plays a crucial role in development by regulating diverse pathways.

### 2.3. Plant Growth, Morphology, and Reproductive Potential of Adult M1 and M2 Plants

Analyzing adult M1 plants harboring ZFN-induced mutations in the NF-YA8 TF uncovered a diverse array of developmental changes. These observations offer novel insights into the function of NF-YA8 in plant growth, adult morphology, and reproductive capacity. Specifically, *nf-ya8-V* mutants exhibited upright growth without side branching and displayed early, synchronized flowering, a desirable trait in tomato breeding. Conversely, *nf-ya8-Y* mutants showed determinate growth, characterized by shorter, sturdier stems and a compact, bushy appearance. The *nf-ya8-A* mutants resembled the wild type in their upright growth. Interestingly, anthocyanin levels, indicators of stress, varied among the mutants. Highly vigorous *nf-ya8-V+* plants showed no anthocyanin production, suggesting NF-YA8′s involvement in stress response. *nf-ya8-S* mutants exhibited compacted growth and abundant flowering, implying NF-YA8′s role in balancing vegetative and reproductive growth. Finally, *nf-ya8-T* mutants with reclining stems and indeterminate growth hinted at NF-YA8′s influence on stem flexibility and growth orientation.

**Figure 5:**
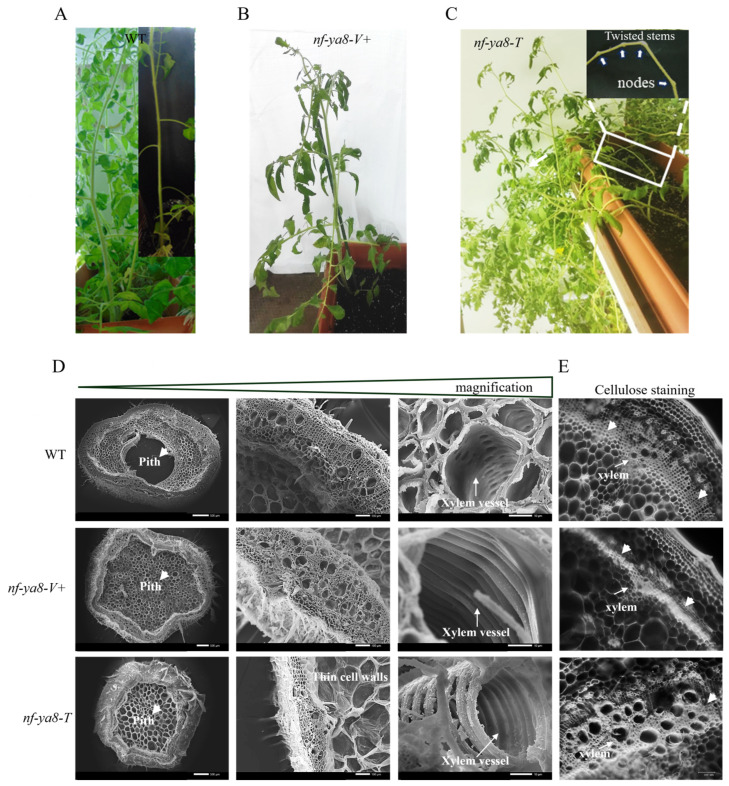
The stem characteristics and anatomical features of extreme *nf-ya8* mutant phenotypes. (**A**) The typical upright growth of the wild type (WT). (**B**) The *Very vigorous* mutant category with thick and upright stems. (**C**) The *Twisted*–reclining stems mutant phenotype with indeterminate growth. The anatomy and cellulose deposition in the upright and reclining stems of *nf-ya8* mutant plants. (**D**) Cross sections of the fourth internode of plants from the wild type (WT) and newly developed M1 *nf-ya8-V*+ and *nf-ya8-T*. Scale bars are 500 µm (first column), 100 µm (second column), and 10 µm (third column). Microscopy images were taken using scanning electron microscopy (SEM) to observe the pith and vascular tissue in both WT and *Very vigorous* (*nf-ya8-V+*) and *Twisted* (*nf-ya8-T*) M1 plants. (**E**) Histochemical staining of the same stems using fluorescent Congo red dye to visualize cellulose distribution and highlight the differences between the WT and *Very vigorous* and *Twisted nf-ya8* mutant plants. Scale bars = 100 µm.

Next, we investigated the stem characteristics and anatomical features of plants exhibiting extreme *nf-ya8* mutant phenotypes (Figure 5A–C). Specifically, we compared *nf-ya8* mutants displaying either upright stems (designated *nf-ya8-V+* and characterized by robust stems) or reclining stems (*nf-ya8-T*) to wild-type plants, using SEM to analyze stem structure (Figure 5D). SEM images revealed significant differences in pith structure. Wild-type stems lacked pith in the center, while *nf-ya8-V+* and *nf-ya8-T* mutants retained pith, suggesting that NF-YA8 affects pith development. Further examination of the *nf-ya8-T* mutants revealed larger pith cells with thinner cell walls and disrupted xylem vessels with decreased lignification, highlighting the importance of lignin for structural support and xylem function.

Histochemical staining with fluorescent Congo red dye, which detects cellulose (Figure 5E), further revealed differences in cellulose distribution. Wild-type plants showed a uniform cellulose layer, while *nf-ya8-V+* mutants displayed increased cellulose deposition in the cambial zone. This suggests that disrupting NF-YA8 directly influences cellulose production and deposition, altering stem growth and development. In conclusion, investigating stem structure and cellulose production in *nf-ya8* M1 plants has provided insights into plant growth and the stem mechanisms that counteract *NF-YA8* disruption. Alterations in pith and cellulose arrangement underscore the TF’s vital function in these mechanisms.

Detailed microscopic analysis of the M2 generation *nf-ya8* disruption mutants revealed that NF-YA8 plays a vital role in tomato trichome development (Figure S1A). We observed significant differences in trichome density and length on the leaf edges of the mutants. Specifically, *nf-ya8-V* and *nf-ya8-V+* mutants consistently displayed short trichomes, albeit at varying densities, while *nf-ya8-T* mutants exhibited trichomes of medium length. Interestingly, *nf-ya8-A* mutants developed glandular trichomes along the leaf edge, unlike the wild type. The *nf-ya8-S* and *nf-ya8-Y* mutants, however, showed a mixture of short and medium and short and long trichomes, respectively, further supporting a role for NF-YA8 in trichome cell differentiation. Furthermore, microscopic examination of anthocyanin accumulation in the underside veins of leaves (Figure S1B,C) revealed variations between the *nf-ya8* mutants and the wild type, suggesting differences in anthocyanin levels. The *nf-ya8-A* mutants exhibited high anthocyanin accumulation, while *nf-ya8-V+* and *nf-ya8-T* mutants completely lacked anthocyanin. We also observed variable anthocyanin accumulation in the stems of both the wild type and the M2 generation *nf-ya8* plants. These marked differences in anthocyanin levels in the *nf-ya8* mutants underscore the TF’s importance in anthocyanin biosynthesis.

Approximately 33% of the *nf-ya8* M1 plants produced fertile offspring, transmitting various NF-YA8 mutations to the M2 generation. These mutations introduced novel traits, demonstrating the effectiveness of ZFNs in functional genomics and tomato breeding. Phenotyping the mature mutant plants revealed phenotypes that might have been missed earlier and highlighted the diverse impact of NF-YA8 on plant growth. Early flowering was also observed in 15% of *nf-ya8-V* category M1 and M2 mutant lines. Reduced flower pedicel length in some M1 plants correlated with fruit setting failure; however, most M2 mutant lines displayed longer flower pedicels and successful fruit setting.

**Figure 6:**
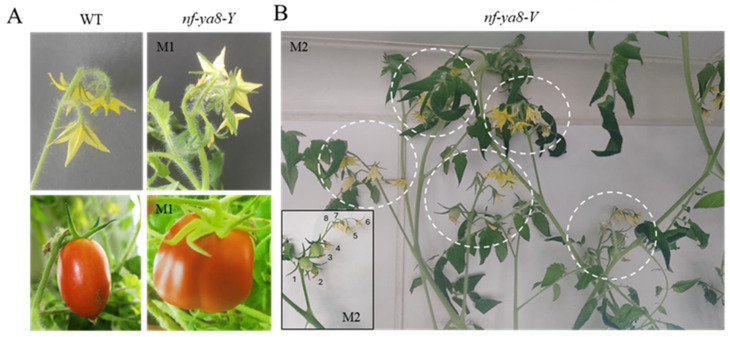
*NF-YA8* disruption leads to changes in inflorescence architecture and fruit morphology. (**A**) The inflorescence architecture of both the wild type (WT) and *nf-ya8-Y* M1 is depicted, along with alterations in fruit size and shape. (**B**) The M2 offspring of *nf-ya8-V* displays abundant and synchronized blooming, with a greater number of florets (8 in the inset) compared to the WT (5).

In the M1 generation, the disruption of NF-YA8 significantly altered inflorescence architecture (Figure 6). Specifically, *nf-ya8-Y* M1 plants showed a substantial increase in the number of florets compared to wild-type plants, along with larger, differently shaped fruits (Figure 6A). This demonstrates a clear link between NF-YA8 and the development of floral organs. Furthermore, *nf-ya8-V* M2 mutant lines displayed a higher degree of synchronized blooming compared to the wild type, characterized by an increased number of florets per inflorescence and per plant (Figure 6B), reaching an average of eight florets compared to five in the wild type. The data suggest NF-YA8 is involved in regulating flowering time and synchronizing floral development. More broadly, this TF demonstrates a pleiotropic function, affecting growth habit, flowering time, stem structure, trichome development, and anthocyanin biosynthesis.

### 2.4. Tomato Fruit Phenotyping

**Figure 7:**
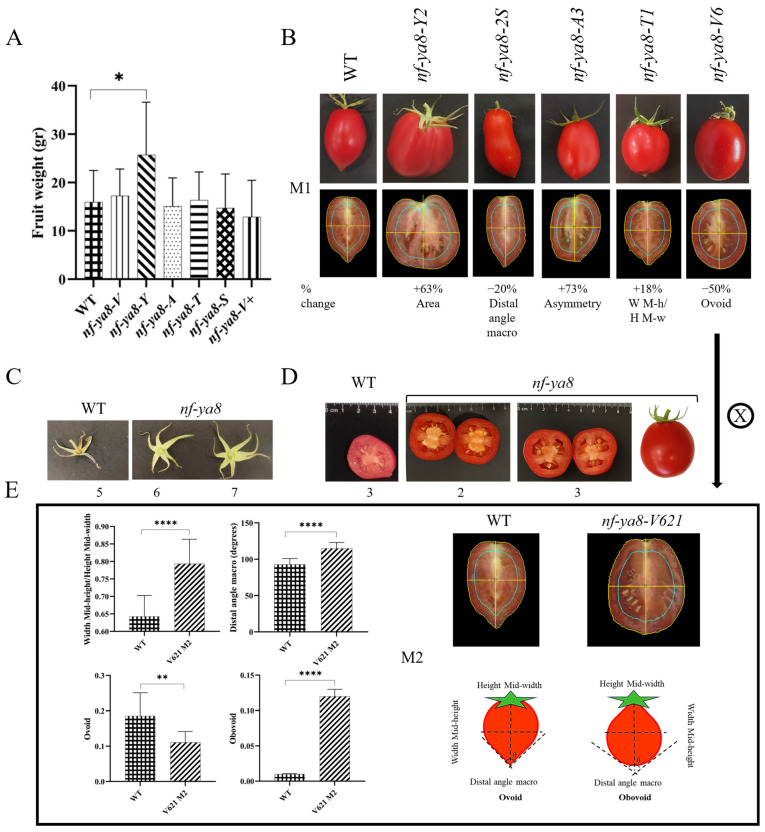
The disruption of *NF-YA8* affects the weight, size, and shape of tomatoes. (**A**), The fruit weight (gr) of the *nf-ya8* M1 generation shows variation, with the *nf-ya8-Y* plants having a significantly greater weight compared to the wild type (WT). Data are presented as mean ± SD (*n* = 6–17), with statistical significance determined by one-way ANOVA. followed by Tukey’s post-hoc test to evaluate pairwise differences between groups. Significance is denoted by asterisks, with * *p* < 0.05. (**B**), Tomato fruit morphometrics in *nf-ya8* M1 mutants showing differences in size (Area) and shape (Digital angle macro, Asymmetry, Width Mid-height/Height Mid-width, Ovoid) descriptors. (**C**), Sepal number also varies in the *nf-ya8* M1 generation compared to the wild type (WT). (**D**), The M2 generation of *nf-ya8* mutants shows variation in locule number compared to the wild type (WT). (**E**), A detailed analysis of the *nf-ya8-V621* fruits in the M2 generation shows significant changes in shape descriptors (Width Mid-height/Height Mid-width, Digital angle macro, Ovoid, Obovoid), indicating the adoption of an obovoid shape with a round bottom, which is not the typical shape of Heinz 1706 tomatoes. W M-h/H M-w: width mid-height/height mid-width. Data are represented as means ± standard deviation (SD); *n* = 12–23. The differences between the groups were determined using Student’s *t*-test. Statistically significant differences are indicated by asterisks: * *p* < 0.05, ** *p* < 0.001, and **** *p* < 0.0001.

Disruption of the *NF-YA8* gene in tomatoes significantly increased fruit size and altered shape (Figure 7).

In the M1 generation, *nf-ya8-Y* plants displayed a notable 1.5-fold increase in fruit size (Figure 7A), and while other mutant groups also showed larger fruits, variation remained high. The M2 generation saw over 25% of fruits exceeding 33 g, nearly double the weight of the wild type. Notably, *nf-ya8-V621* plants had an average fruit weight 43% greater than the wild type. Digital phenotyping enabled detailed analysis of external and internal fruit qualities, revealing variations in size, shape, asymmetry, and color (Figure 7B). Specifically, the M1 generation *nf-ya8-Y* plants demonstrated a 63% increase in fruit area compared to the wild type. Other M1 mutant categories also showed distinct shape differences: *nf-ya8-S* displayed a 20% decrease in distal angle macro, *nf-ya8-A* a substantial 73% difference in asymmetry, *nf-ya8-T* an 18% increase in the ratio of mid-height to height mid-width, and *nf-ya8-V* a 50% decrease in ovoid shape. Unlike wild-type Heinz 1706 with its typical five sepals, many *nf-ya8* mutant plants developed 6 or 7 sepals (Figure 7C).

M2 generation mutants also varied in locule number, showing two or three instead of the wild type’s three (Figure 7D). The *nf-ya8-V621* M2 generation exhibited a 23% increase in the ratio of mid-height to height mid-width, a 24% increase in distal angle macro, and approximately 50% of the tomatoes in this category showed a 40% decrease in the ovoid shape, while the remaining 50% displayed a transition from ovoid to obovoid shape. Furthermore, *nf-ya8-V621* fruit displayed a 5% expansion in mid-width and a 17% decrease in length compared to the wild type (Figure 7E), resulting in a more desirable, rounder shape at the distal end, a trait beneficial for industrial-type tomatoes like Heinz 1706 because pointed tips can compromise fruit quality and increase susceptibility to bacterial infection during transportation. This increased size and weight observed in the mutants could potentially translate to higher yields and increased profitability for farmers.

## 3. Discussion

ZFNs, characterized by their modular architecture incorporating ββα motifs, have proven to be a valuable tool for targeted genome editing in a variety of plant species, including tomato, tobacco, soybean, *Arabidopsis*, rice, and wheat [17]. By using ZFNs to edit the genome of tomato seeds without introducing foreign DNA, we successfully targeted the *NF-YA8* gene near the DBD. The ability of the TFs to bind specific DNA sequences through DBD domains allows them to orchestrate gene expression programs critical for survival and reproduction. Consequently, mutations affecting DBDs can have dramatic and often deleterious effects on plant phenotypes. Building upon our earlier findings that the disruption of the NF-YB subunit L1L4 near DBD leads to significant phenotypic changes [4] and fruit characteristics [5], we hypothesized that a similar disruption of the NF-YA8 subunit will also result in notable developmental consequences. Indeed, this disruption resulted in diverse mutations within the gene, which led to a range of phenotypic variations affecting both the vegetative and reproductive stages of the plant. These pleiotropic effects on embryo development (including cotyledon formation), seedling growth, stem structure, flowering, and fruit development highlight the high-level regulatory role of the NF-YA8 TF in tomato plant development. While we observed preferential inheritance of some mutations, further purification of mutant lines is necessary to fully understand the underlying genetic mechanisms. Other examples highlight the critical role of DBDs in proper plant development and response to environmental cues. Specifically, mutations in the B3 DBD of the ABI3 and VP1 TFs disrupt their ability to bind DNA and regulate gene expression, leading to severe developmental defects in plants [18,19]. Disrupted ABI3 function results in precocious germination, reduced desiccation tolerance, and impaired seed dormancy [18]. Similarly, mutations in the VP1 B3 domain cause vivipary, the premature germination of seeds on the cob. Both examples highlight the importance of functional DBDs for proper plant development and response to environmental cues [19]. This study further highlights the importance of understanding the structural consequences of mutations in NF-YA8 TF. By employing homology modeling and structural analysis, we have gained valuable insights into how mutations near the A1 and A2 helices can potentially affect NF-Y complex formation and DNA binding, leading to altered gene expression and phenotypic changes in tomato.

This study further examined the location and possible origins of the observed mutations. Results indicate high ZFN specificity, with a high frequency of on-target mutations, mostly in exon 6 (positions 692–935) and some in exon 5. These mutations, including indels, often lead to frameshifts and loss of gene function, demonstrating the potential of ZFNs for efficient genome editing. However, the identification of linked mutations in both exon 2 and exon 6, albeit in a small proportion of mutant plants (2.85%, 2/70), underscores the need for careful evaluation of potential off-target activity. The fact that these linked mutations resulted in severe phenotypic abnormalities and non-viability underscores the functional importance of the regulatory domain in exon 2 and the DNA-binding domain in exon 6 of the TF. This finding suggests that disrupting the regulatory domain in exon 2, in conjunction with the disruption of exon 6, leads to a synergistic detrimental effect on gene function. We propose that chromatin loop formation provides a plausible mechanistic explanation for the observed linked mutations. Chromatin loops are three-dimensional structures formed by the folding and looping of chromatin, bringing distant regions of the genome into close spatial proximity [20]. In this scenario, the spatial proximity between exon 2 and exon 6 could facilitate ZFN cleavage at both sites either simultaneously or rapidly sequentially. The subsequent repair of the DSBs via non-homologous end joining (NHEJ) could then lead to the introduction of mutations at both exon 2 and exon 6. Ultimately, any undetected changes can be removed via traditional breeding. Natural selection allowed mutations that had a reproductive advantage to pass in the M2 generation.

This ZFN editing technology allowed for precise and reproducible genetic modifications, which resulted in phenotype groups, early in development, leading to a range of mutant traits related to growth, flowering, and fruiting.

TFs act as molecular switches, turning genes “on” or “off” in response to developmental signals, environmental cues, and hormonal stimuli, and the disruption of their function can have profound consequences.

The *nf-ya8* M2 lines displayed a diverse range of alleles due to the parental plant’s chimeric nature, leading to complex heterozygous genotypes. Analyzing later generations (M3 onwards) will allow for a more precise understanding of the mechanisms behind ZFN-induced mutations and their effects on the plant’s traits. Despite the mutations, the core domain of the NF-YA8 protein, crucial for trimeric complex formation, remained highly conserved. As expected, mutations were frequently observed near the ZFN binding site in exon 6. The less frequent mutations found in exon 2, distant from the binding site, could potentially be explained by DNA looping mediated by the FokI domain of the ZFN. Mutations in the tomato gene *SlNF-YA8* lead to a range of developmental abnormalities, including both stunted and vigorous plant growth, altered apical dominance, misshapen cotyledons, and changes in anthocyanin production. These diverse phenotypes emphasize *SlNF-YA8*′s crucial role in regulating fundamental plant development. *SlNF-YA8* shares significant sequence similarity with *Arabidopsis NF-YA* homologs (31.17% with *AtNF-YA5*, 30.49% with *AtNF-YA8*, and 27.27% with *AtNF-YA3*), and in *Arabidopsis*, *AtNF-YA3* and *AtNF-YA8* are essential for early embryo development, with their knockdown resulting in defective embryos and impaired auxin responses. Similarly, tomato *nf-ya8-S* seedlings exhibit a heritable dwarf phenotype, consistent with observations in *Medicago truncatula*, where *nf-ya1* mutants also display dwarfism. This aligns with the broader understanding of the NF-YA transcription factor family, which is known to influence various aspects of plant development. For example, mutations in other *NF-YA* genes, such as *nf-ya1*, *nf-ya2*, and *nf-ya10*, have been linked to reduced leaf size and dwarfism. Interestingly, *SlNF-YA8* expression is upregulated upon *Argonaute1 (AGO1)* silencing in tomato. In our study, *nf-ya8-T* mutants show delayed flowering, which parallels the established role of NF-YA proteins in promoting flowering via FLOWERING LOCUS T in *Arabidopsis*.

Plant architecture, a key determinant of agricultural productivity, is governed by factors like branching patterns, internode length, and shoot determinacy [21]. In tomatoes, the *SELF-PRUNING* (*SP*) gene plays a central role by regulating flowering and influencing shoot termination via antiflorigen suppression [22]. This mechanism has been exploited to develop determinate tomato varieties suitable for mechanized harvesting due to their compact growth habit and lack of apical dominance. Mutations in *SP* can lead to variations in plant compactness and overall yield [23]. Similarly, the *NF-YA8* gene significantly impacts stem phenotypes, leading to diverse architectures ranging from upright to reclining stems and varying degrees of branching, highlighting its role in controlling plant architecture and generating horticultural diversity. For instance, the *nf-ya8-T* mutant exhibits a distinctive reclining stem phenotype, while other *nf-ya8* mutants display upright growth. Considering the established role of polar, basipetal auxin transport in apical dominance [24] and anatomical evidence indicating *NF-YA8*′s influence on pith growth and vascular development, our research suggests that *NF-YA8* may regulate auxin signaling by influencing gene expression related to cell division and elongation within the pith, ultimately affecting stem thickness. Furthermore, we found that *NF-YA8* modulates both cellulose biosynthesis and its distribution in tomato stems, factors crucial for stem strength and stability. Auxin maintains stem stability by promoting cell division and elongation in the lateral meristem, and given its transport within the vascular parenchyma and effect on the vascular cambium [25], *NF-YA8*′s potential to control auxin signaling via gene expression is strongly implied. Additional research is warranted to fully elucidate the intricate relationship between *NF-YA8* and auxin signaling during shoot development. The altered xylem vessels and cellulose distribution observed in *nf-ya8* mutants emphasize the critical role of the *NF-YA8* TF in these processes. Cellulose, a key cell wall component vital for plant growth [26], and xylem structure are both known to affect plant development [16,27]. Our study identifies *NF-YA8* as a novel regulator of cellulose content and xylem structure in tomato. Interestingly, elevated expression of *NF-YA2* has been reported in *Dendrobium catenatum* stem tissue [28], mirroring findings in *Brassica napus* where stems exhibit high *NF-YA3* expression [29]. The reclining growth habit observed in *nf-ya8-T* stalks may be adaptive for resource and sunlight acquisition in dense populations and is associated with modifications to lignocellulose, which provides strength and rigidity. Future studies should also investigate the interactions between *NF-YA8* and other hormones.

Our gene expression results demonstrated that disruption of *NF-YA8* leads to significant alterations in gene expression, resulting in a spectrum of phenotypic variations in tomato seedlings. The dramatic upregulation of *CHS2* and *F3H* in the Anthocyanin and Twisted mutants provides a clear link between *NF-YA8* and the anthocyanin biosynthesis pathway. This suggests that *NF-YA8* may normally act as a repressor of anthocyanin production, and its disruption leads to the derepression of these key biosynthetic genes. The increased expression of *FW2.2* in the Vigorous and Ypsilon mutants suggests a role for *NF-YA8* in regulating fruit development even at the seedling stage. *FW2.2* is a well-known regulator of fruit weight, and its upregulation in these mutants may contribute to altered cell division patterns and growth characteristics. The variable expression of hormone-related genes (*GA20OX1*, *ABI3*, *ETR1*, *IAA*) in the different mutant backgrounds suggests that *NF-YA8* also plays a role in hormone signaling pathways. Gibberellins (GAs) are involved in stem elongation, and the increased expression of *GA20OX1* in the Twisted and Ypsilon mutants may contribute to their altered morphology. Similarly, ABA, ethylene, and auxin signaling pathways are known to regulate various aspects of plant development, and the altered expression of *ABI3*, *ETR1*, and *IAA* in the different mutants suggests that *NF-YA8* may modulate these pathways to influence growth and development. The upregulation of *IAA* in the Slow growth mutant may be compensating for growth reduction, which is a survival response. The finding that *L1L4* expression was also altered in the *nf-ya8* mutants is interesting. It suggests that NF-YA8 might influence the expression of other NF-Y subunits, possibly affecting the formation or activity of the entire NF-Y complex. This could have broader implications for NF-Y-mediated transcriptional regulation. The role of *NF-YA8* in regulating plant architecture is supported by the mutant phenotypes.

Microscopic analysis of the M2 generation *nf-ya8* mutants revealed altered leaf trichome morphology. Trichomes, epidermal cell structures present on aerial plant surfaces, protect against herbivores and pathogens, and regulate water loss and temperature [30]. Tomato, a member of the *Solanaceae* family abundant in trichomes, boasts seven distinct trichome types that vary in length and branching patterns, with types I and VI possessing notable glandular tips [31]. Our findings demonstrate a significant role for the NF-YA8 TF in tomato trichome development and morphology. Compared to wild-type plants, *nf-ya8* mutants exhibited altered trichome characteristics, suggesting a novel function for SlNF-YA8 in regulating cell differentiation within tomato trichomes. Other studies support the importance of NF-Y subunits in trichome development; for example, disruption of NF-Y subunit B6 L1L4 leads to changes in trichome density and morphology [4], and silencing *Argonaute1 (AGO1)* impacts trichome development and increases *SlNF-YA8* expression [32]. In *Arabidopsis*, mutation of *LEAFY COTYLEDON1 (LEC1)* causes abnormal trichome growth [33], highlighting the conserved role of NF-Y TFs in regulating this process across plant species. Future research investigating the downstream regulatory targets and interactions of NF-YA8 with other TFs will provide a deeper understanding of the molecular mechanisms governing trichome formation in tomato.

This study revealed the significant role of NF-YA8 in controlling anthocyanin production, especially within leaf veins. We found that the *nf-ya8-A* mutants exhibited elevated levels of anthocyanins, notably in the leaf veins, whereas the *nf-ya8-V+* mutants showed an absence of this accumulation. This vein-specific anthocyanin accumulation is known to protect against environmental stressors like UV radiation, drought, and high temperatures [34]. Consistent with these findings, *NF-YA5*, which is highly expressed in leaf tissues, including the vasculature, has also been linked to anthocyanin regulation [35]. Similarly, our previous research demonstrated that the disruption of *L1L4* in tomato led to anthocyanin accumulation in the hypocotyls following brief light exposure [4].

Plant reproduction is fundamentally important, and the NF-Y TF complex appears to play a crucial role in this process in tomatoes. Disruption of the *NF-YA8* gene caused sterility in 66% of M1 plants, a higher rate than the 50% sterility observed with *L1L4/NF-YB6* disruption [4], suggesting a greater importance for the NF-YA subunit in reproductive success. Interestingly, some fertile *nf-ya8* mutants exhibited altered flowering characteristics: 15% of *nf-ya8-V* mutants flowered two months earlier than wild-type plants, and M2 offspring displayed an increased number of florets per inflorescence. This aligns with previous research in *Arabidopsis*, where *NFYA5* regulates flowering time and senescence [34], and *NF-YB2* and *NF-YB3* promote flowering additively [36]. Tomato *FRUITFULL*-like genes, *FUL2* and *MBP20*, are also known to be essential determinants of the transition to reproductive growth, regulating inflorescence branching and floral meristem maturation [37]. Understanding the genetic mechanisms governing fruit development is crucial for enhancing both plant reproduction and agricultural yields, making it a key target for crop improvement. In this study, we investigated the role of the *NF-YA8* gene in tomato fruit development and found that it significantly influences fruit size and shape. Specifically, disrupting *NF-YA8* resulted in substantial alterations to fruit morphology, including changes in weight, dimensions, overall shape, sepal number, and fruit angle ratios.

## 4. Materials and Methods

### 4.1. Plant Material and Growth Conditions

The C. M. Rick Tomato Genetics Resource Center in Davis, CA, USA, provided *S. lycopersicum* (cv. Heinz 1706) seeds. The plants were grown in pots under controlled conditions of 25 °C and a photoperiod of 16 h light and 8 h darkness.

### 4.2. Chemicals

EvaGreen^®^ Dye for high-resolution melting (HRM) was purchased from Biotium, Inc. (Fremont, CA, USA). Congo red was purchased from UNI-CHEM Chemical Reagents (Haw River, NC, USA).

### 4.3. Target Site Selection and ZFN Pair Design

The target region is composed of a pair of half-sites consisting of nine base pairs (bps) each, namely the left half-site (5′-GCTCTACCC-3′) and the right half-site (5′-AGGACGCTT-3′). These half-sites are recognized by three zinc finger arrays, with the three fingers of the left ZFP having the following recognition helices: Finger 1 (triplet GCT): TSGELVR; Finger 2 (triplet CTA): QNSTLTE; Finger 3 (triplet CCC): SKKHLAE. To generate the amino acid sequence for the left ZFP, a combination of preset sequences was utilized, along with the recognition helices. The N-terminus was held constant with the cloning sequence "LEPGEKP", while the backbone of the N-terminus was "YKCPECGKSFS". The C-terminus backbone of the left ZFP contained the sequence "HQRTH", and the ZF linker had the sequence "TGEKP", while the C-terminus was anchored with the cloning sequence "TGKKTS". These sequences, along with the recognition helices, were used to construct the amino acid sequence for the left ZFP. On the other hand, the right ZFP was composed of three fingers, which identified the 9 bp right half-site 5′-AGGACGCTT-3′. The recognition helices for the right half-site were as follows: Finger 1 (triplet CTT): TTGALTE; Finger 2 (triplet ACG): RTDTLRD; Finger 3 (triplet AGG): RSDHLTN. Further sequences included the N-terminus, which was fixed with the cloning sequence "LEPGEKP", and the backbone of the N-terminus was "YKCPECGKSFS". The C-terminus backbone for the right ZFP was "HQRTH" and the ZF linker was "TGEKP", with the C-terminus anchored with the cloning sequence "TGKKTS". These sequences were utilized in conjunction with the recognition helices to construct the amino acid sequence for the right ZFP. The DNA sequences encoding the two zinc finger arrays were identified by Zinc Finger Tools and were optimized by using *S. lycopersicum* DNA codon bias and convenient XbaI (5′) BamHI (3′) restriction sites for subcloning. The sequences encoding the two three-finger ZFP arrays were further optimized to prolong the half-life of the mRNA, using as a criterion the Codon Adaptation Index (CAI). These sequences were then synthesized by Genscript and ligated into the pUC57 plasmid, with verification through sequencing. The next step involved digesting the DNA fragments encoding the ZFPs with XbaI and BamHI and inserting them into the pDW1775 ZFN expression vector (plasmid 13456) provided by Addgene. This vector is a binary vector containing a promoter region and a selectable marker gene for expression in plants. The ZFP coding sequences were cloned into the pDW1775 vector using the standard molecular cloning protocol. The resulting constructs, L and R, were then transformed into *E. coli*, and the recombinant clones containing the ZFN coding sequences were isolated, and their plasmids extracted.

### 4.4. Transient Expression of ZFNs in Tomato Seeds

Initially, the surfaces of the seeds were sterilized to eliminate any potential presence of bacteria or fungal spores. This was achieved by submerging them in a 70% ethanol solution for 20 s, followed by rinsing with autoclaved deionized water. To further disinfect the seeds, they were then immersed in a 3.5% sodium hypochlorite solution for 10 min. Any remaining traces of sodium hypochlorite were removed by rinsing the seeds with autoclaved water. Next, the seeds were placed in a germination buffer consisting of 5% sucrose, 3% H3BO3, and 1.3 mM Ca (NO_3_) to promote germination before undergoing electroporation treatment. To facilitate this process, the seeds were kept in the dark at 10 °C for 12 h. The mature tomato seeds were transformed through electroporation by direct transfer of the ZFN encoding genes as described previously [38]. Briefly, the seeds were washed with sterilized water and moved into the electroporation buffer, consisting of 80 mM KCl, 5 mM CaCl2, 10 mM Hepes, and 0.5 M mannitol at a pH of 7.2. This specific buffer was carefully formulated to create an ideal environment for electroporation while ensuring the viability of the seeds. Each sterile electroporation cuvette (obtained from Sigma-Aldrich, St. Louis, MI, USA) was filled with nine germinating seeds and 200 μL of the electroporation buffer, along with 50 μg of freshly prepared and well-mixed plasmid DNA (25 μg for each ZFN monomer). To eliminate any air bubbles and guarantee full submersion of the seeds in the buffer, the cuvettes were placed in a vacuum container at 0.09 MPa for 10 min. Afterward, the cuvettes were left at room temperature for 1 h, followed by a 10 min stay on ice, before the actual electroporation process. The seeds were subjected to three pulses of 4 milliseconds each, using a commercial electroporation device (MicroPulser, Bio-Rad, Hercules, CA, USA), at a field strength of 6.25 KV cm-1. Upon completion of electroporation, the seeds were left on ice for an additional hour and then placed in Petri dishes containing the electroporation solution for 24 h at 10 °C in the absence of light. The next day, the seeds were sown in Jiffy pots and kept under a 16 h light cycle at a constant temperature of 25 ± 2.4 °C until transplantation.

### 4.5. Determination of Plant Height and Stem Diameter

The height, diameter, and number of nodes were recorded for tomato plants at the seedling stage of plants of identical age. Measurements for height were in centimeters, while stem diameter was measured in millimeters.

### 4.6. Microscopic Analysis

For histological analysis of stems, Congo red (0.5% aqueous solution) was used to visualize cellulose under ultraviolet light excitation with the aid of a filter with a bandpass of 560/40. A Zeiss Axioskop 40 C/FL microscope (ZEISS, Oberkochen, Germany) was utilized to examine plant specimens, with the assistance of ProgRes Capture Pro acquisition software v2.8.8 and Acroplan (Sugar Land, TX, USA) (5×, 10×) objective lenses. Using scanning electron microscopy (SEM), a thorough investigation was conducted on the stems of tomato plants. Both wild-type and *nf-ya8* M1 mutant stems were manually cut into 1 to 2 cm sections using a razor blade, followed by immersion in 100% methanol for 30 min. After that, the samples were immersed in 100% ethanol for another 30 min and left to dehydrate overnight at 4 °C. To remove any gases from the tissues, the specimens were then treated with a vacuum pump for 15 min. Subsequently, they were coated with gold using an Au Sputter Coater 108 (Agar Scientific, Stansted, UK) and imaged at various magnifications in an electron microscope JSM-IT500 (InTouch Scope; JEOL, Tokyo, Japan).

### 4.7. Digital Fruit Phenotyping

Tomato Analyzer software (version 3.0) was used to analyze longitudinally cut and scanned tomato fruits from both wild-type and mutant plants, with a resolution of 300 dpi. After saving the resulting images in TIF format, they were processed and examined for morphometric and colorimetric characteristics. All fruits from the *nf-ya8* phenotypic category and the wild type were included in the analysis. The software measured various fruit parameters, such as size and shape, including area, width, height, and distal end descriptors such as macro and micro distal angle, ovoid asymmetry, obovoid asymmetry, and vertical asymmetry. The percentage change in tomato fruit descriptors was computed using the equation (m-wt)/wt×100%, with m representing the current value of the mutant fruit and wt representing the value of the wild type.

### 4.8. Detection of ZFN-Induced Modifications at Target Gene

Genomic DNA was extracted from both ZFN-treated and wild-type (control) samples using the NucleoSpin Plant II kit (Macherey-Nagel, Düren, Germany). Similarly, RNA was isolated from the same tissue using the NucleoSpin RNA Plant kit (Macherey-Nagel). Following this, the PrimeScript first strand cDNA synthesis kit (TaKaRa Biomedicals, Otsu, Japan) was used to perform cDNA synthesis.

To detect mutations in a specific location of DNA, the method of PCR analysis was utilized. This technique involved utilizing genomic or cDNA extracted from M1 mature plants and a control wild type as a template for the PCR-based analysis. Each sample’s reaction mixture, measuring 25 μL, contained 20 ng of genomic DNA, 10 mM TRIS-HCl (pH 8.4), 50 mM KCl, 2.0 mM MgCl2, 160 μM of each dNTP, 1.0 U of Kapa Taq polymerase (Kapa Biosystems, Cape Town, South Africa), and 200 nM of specific primers (listed below). The denaturation process began at 95 °C for 2 min, followed by 25 cycles of amplification in a thermal cycler (with 1 min at 94 °C, 1 min at 45 °C, and 1.5 min at 72 °C) and a final extension of 7 min at 72 °C. The amplified DNA products were then separated into 1% (*w*/*v*) agarose TAE gels, stained with ethidium bromide, and visualized using ultraviolet light.

For HRM analysis on M1 plants, primers were designed on the flanking sequence of the NF-YA8 target site: SlNFYA8sF: TCCTCTTGAATGCACCGAGAGCTTGCC and SlNFYA8sR: GAGACTCTGAACTCTGGTTGC. The PCR reactions, DNA melting, and fluorescence level acquisition were performed using the Rotor-Gene 6000 real-time 5P HRM PCR Thermocycler (Corbett Research, Sydney, Australia). The reaction mixture volume was 15 μL, consisting of 20 ng genomic DNA, 1X PCR buffer, 2.5 mM MgCl2, 0.2 mM dNTP, 300 nM forward and reverse primers, 1.5 mM Syto^®^ 9 green fluorescent nucleic acid stain (Life Technologies Corp., Paisley, UK), and 1 U Kapa Taq DNA polymerase (Kapa Biosystems, Cape Town, South Africa). The PCR protocol began with an initial denaturation at 95 °C for 3 min, followed by 35 cycles of denaturation at 95 °C for 20 s, annealing at 60 °C for 20 s, extension at 72 °C for 20 s, and final extension at 72 °C for 10 min. The resulting melting profiles were compared to a horizontal line representing the wild-type sample. Any significant deviations from this line, compared to the wild-type controls, indicated potential sequence changes within the amplicon being analyzed. The DNA amplification products were also visualized using 1% (*w*/*v*) agarose TAE gels stained with ethidium bromide and detected with ultraviolet light. To validate the HRM results, sequencing was performed to confirm the detection of zinc finger nuclease-based mutations.

Sanger sequencing was employed to confirm the effectiveness of targeted mutations generated by ZFN. This involved amplifying the full-length sequences of the target gene from *E. coli* clones derived from ZFN-edited plants. The resulting sequence data were then compared with the wild-type sequence to detect any introduced mutations. Upon amplification of the *NF-YA8* gene from both M1 and wild-type plants, the resulting PCR products were cloned in a directional manner and subsequently inserted into chemically competent *E. coli* cells for propagation. Clones were chosen at random and subjected to colony PCR using NFYAF: ATGCTAAGTTTCTCAAAGAAAGGT and NFYAR: TCAGGTTCCAACATGCAGGAAGTCTTC primers to verify the full cDNA sequence, followed by HRM analysis for identification of any potential mutations. The positive clones were further validated by sequencing. To perform genotyping on the M1s and their offspring, we utilized primers that enabled PCR-based amplification of both full-length cDNA and shorter sequences near the ZFN target site. DNA sequence alignments were visualized using Benchling (www.benchling.com).

### 4.9. Screening for Off-Target Effects

Using bioinformatics tools during ZFN design process, a comprehensive investigation was conducted on the tomato genome to identify any potential off-target sites with high sequence similarity to the target exon. For genome-wide screening of the potential off-target effects of ZFN genome editing in tomatoes, a customized Unix script was developed, which was used in bioinformatic analysis (Supplementary Materials Text S1) and revealed a unique occurrence within the *NF-YA8* coding sequence. Briefly, the custom shell script ZFNsearch.sh was used to find patterns in the specific genome given. The script takes the genome fasta file, two patterns, and a number representing allowed random nucleotides between them as input. It uses grep to extract sequences matching the pattern pair and also extracts a 200 bp region (100 bp upstream and 100 bp downstream) around the matched region. The text below shows the specific pipeline used to generate the commands to execute the script with different pattern combinations, using the text prepared with the script Prepare_Genome.sh to reference the tomato genome SL3.1. The pipeline checked for 56 possible ZFN target sequences, resulting in a single genomic hit, verified further using Blastn. Furthermore, to evaluate the effects of ZFN-*NF-Y8*, we utilized HRM analysis and gel electrophoresis of the PCR-amplified DNA fragments from closely related sequences found in *Solyc01g006930.2.1*, *Solyc01g087240.2.1*, and *Solyc11g065700.1.1*. For *Solyc11g065700.1.1*, we used the primers SlNFYA57F: GCCACAAGAAATGGCTCAAGAA and SlNFYA57R: CTGGTACTCATCCTTTGATTCTTG. For *Solyc01g087240.2.1*, the primers used were SlNFYA24F: GCCTCTCGACATGGAAGAGGA and SlNFYA24R: CGCGGAAAAATACACAGATGAACCATGGCC. And for *Solyc01g006930.2.1*, the primers used were SlNFYA93F: GAATTTGGCTTCTGATGAAGG and SlNFYA93R: CAGGTTTTGGAACAGAAAAGGATC. The sequences were retrieved from the Ensembl Plants (https://plants.ensembl.org/index.html, accessed on 10 February 2022) browser.

### 4.10. Gene Expression Analysis

For gene expression studies on synthesized cDNA from the different genotypes (wild-type, mutant lines), quantitative RT-PCR reactions were performed in a 20 μL volume using 1 μL of the diluted cDNA as a template, Phusion Hot Start DNA Polymerase (New England BioLabs, Ipswich, MA, USA), and gene-specific primers of known genes and tomato sequences retrieved from publicly available resources. A set of 11 genes was selected for gene expression analysis in tomato seedlings, encompassing *EF1-α*, *NF-YA8*, *CHS2*, *F3H*, *FW2.2*, *UBI*, *NF-YB*/*L1L4*, *ABI3*, *ETRI*, *GA20OX1*, and *IAA*. Primer sequences were largely based on a previously published study [4]. However, the primer sequences for *F3H* (*Solyc02g083860*; F: ATGGCACCTTCAACACTAACAG, R: TTAAGCAAGAAT TTCCTCAATGGG) and *NF-YA8* (*Solyc08g062210*; F: ATGCTAAGTTTCTCAAAGAAAGGT, R: GGTTCCAACATGCAGGAAGTCTTC) were specifically designed and used in this study. For qPCR, each sample reaction was set up in a PCR reaction mix (20 μL), containing 2× Phusion Hot Start Flex 2X Master Mix (New England BioLabs), 0.2 mM forward and reverse primers, 1× EvaGreen^®^ dye (Biotium, Fremont, CA, USA), and 1 μL of the diluted cDNA. qRT-PCR reactions were performed in a Corbett Rotor Gene 6000 Thermocycler (Corbett Research, Sydney, Australia). The PCR-based DNA amplification conditions were as follows: (i) an initial denaturation step at 98 °C for 3 min, (ii) an amplification step with 35 cycles at 98 °C for 30 s, 55 °C for 50 s, 72 °C for 30 s, and (iii) a final elongation step at 72 °C for 10 min. Gene expression levels were normalized to *EF1-α* (*Solyc06g005060*) expression in each genotype. The relative fold change in expression was then calculated using the 2^−∆∆CT^ method [39], with the wild-type genotype serving as the calibrator. All qPCR data were plotted using GraphPad Prism version 9. Gene expression data are expressed as the mean ± SE of three biological replicates, and technical replicates were also performed. Agarose gel electrophoresis was used to visualize the amplicons.

### 4.11. In Silico Protein Structure Analyses

Utilizing the amino acid sequences as a starting point, SWISS-MODEL (https://swissmodel.expasy.org, accessed on 10 January 2024) was employed to predict the most likely three-dimensional quaternary structures of the wild-type and mutant proteins of NF-YA8. The resulting PDB files were generated using SWISS-MODEL [40], based on the AlphaFold model template structure with the best fit. Additionally, a MultiSeq analysis [41] within the Visual Molecular Dynamics (VMD) software [42], version 1.9.4 was utilized to merge sequence and structure data, facilitating bioinformatics assessments.

### 4.12. Statistical Analyses

The data were analyzed using GraphPad Prism 6.0 software (GraphPad, San Diego, CA, USA). ANOVA was used to determine if there were any significant (*p* < 0.05) differences among groups means at *p* < 0.05, followed by Tukey’s post-hoc test for multiple comparisons to determine which means differ significantly. The differences between the means of groups were determined using Student’s *t*-test. Two-way ANOVA was employed to statistically analyze the relative gene expression data (fold change), evaluating the main effects of gene and genotype, as well as their interaction.

## 5. Conclusions

This study demonstrates that NF-YA8 is crucial for plant growth and development. The diverse phenotypes of *nf-ya8* mutants suggest its role in multiple developmental pathways, warranting further investigation into its mechanism of action and interactions with other factors. Changes in fruit and sepal morphology emphasize NF-YA8′s impact on tomato development. The round fruit phenotype of *nf-ya8-V621* offers breeding potential for improved tomato fruit shape and reduced spoilage. These findings may extend to other crops, enabling genome editing strategies for crop enhancement.

## Data Availability

Data are contained within the article and the Supplementary Materials.

## Supplementary Materials

The following supporting information can be downloaded at https://www.mdpi.com/article/10.3390/plants14121826/s1, Figure S1: The trichome phenotypes and anthocyanin accumulation of the wild-type and *nf-ya8* mutants; Text S1: Genome-wide scanning for putative off-target sites. Reference [43] is cited in the supplementary materials.
