# Supplementary material for: Genome Editing of the NF-YA8 Gene Modifies Tomato Plant Architecture and Fruit Traits
Source: Plants (Basel). 2025 Jun 13;14(12):1826. doi: 10.3390/plants14121826 (PMC12196555; doi:10.3390/plants14121826)
Supplement: Supplementary file 1 [file plants-14-01826-s001.zip › Figure S1.pdf]

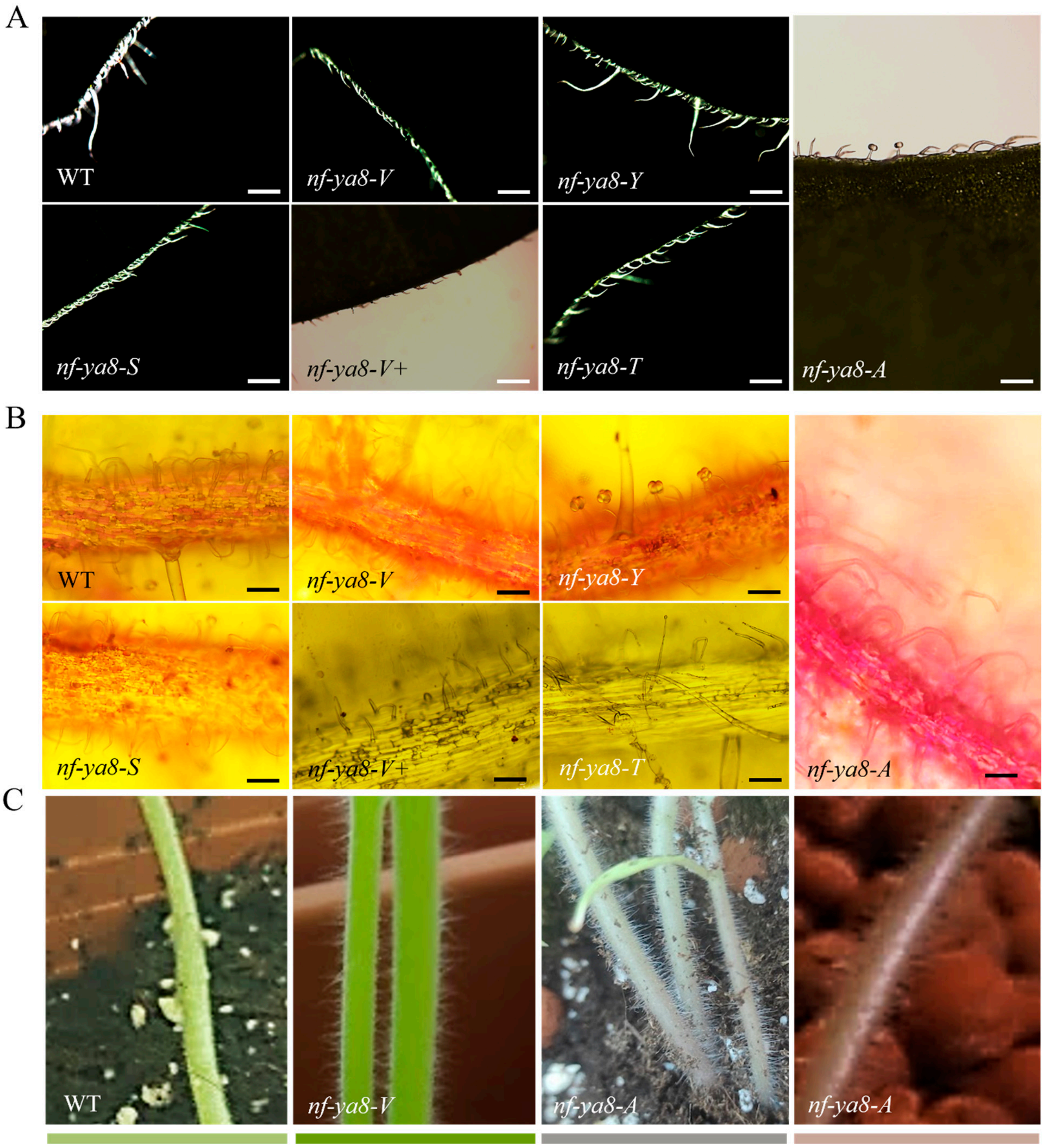

Figure S1. The trichome phenotypes and anthocyanin accumulation of the wild-type and *nf-ya8* mutants. (A), The trichome phenotypes of the M2 generation compared between the wild-type (WT) and *nf-ya8* lines examined using light microscopy. (B), Anthocyanin accumulation on the leaf veins of the abaxial side in wild-type (WT) and *nf-ya8* mutant plants at 5X magnification. (C) Anthocyanin accumulation in the stems of wild-type (WT) and M2 generation *nf-ya8* plants. Scale bars=50μm.
