# Supplementary material for: Genome Editing of the NF-YA8 Gene Modifies Tomato Plant Architecture and Fruit Traits
Source: Plants (Basel). 2025 Jun 13;14(12):1826. doi: 10.3390/plants14121826 (PMC12196555; doi:10.3390/plants14121826)
Supplement: Supplementary file 1 [file plants-14-01826-s001.zip › Text S1.pdf]

TEXT S1

Genome editing of the *NF-YA8* gene modifies tomato plant architecture and fruit traits

Nestor Petrou , Nikoleta Tsigarida , Zoe Hilioti

Genome-wide scanning for putative off-target sites

To thoroughly evaluate the potential off-target effects of Zinc Finger Nuclease (ZFN) genome editing in tomatoes, a customized Unix script, named ZFNsearch, was developed. This script harnesses the flexibility of Unix scripting to explore a comprehensive range of parameters in conjunction with other analysis methods, such as the ZFN-Site tool (https://epd.expasy.org/tagger/targetsearch.html). Initially, the ZFN-Site tool was employed to generate putative starting off-target sites with a maximum spacing of 5-6 base pairs between ZFN half-sites and a maximum of two mismatches per half-site. Furthermore, the ZFNsearch script enables the specific systematic scanning of the entire tomato genome (RefSeq: GCF_000188115.5) to identify potential off-target sites. Later, the script incorporated filtering based on ZFN target recognition, considering variability in the distance between the two ZFN half sites (3 to 10 base pairs) and allowing for mismatches. Furthermore, the script integrated flanking sequences around the ZFN half sites, enabling the utilization of existing bioinformatics pipelines (Blastn; Altschul et al., 1997) to efficiently process and analyze potential off-target hits (https://github.com/ikaramichali/ZFNsearch). The searched ZFN sites are shown below:

AGCTAGGGG n(X3 to 10) AGGACGCTT
AAGCGTCCT n(X3 to 10) CCCCTAGCT
AAGCGTCCT n(X3 to 10) AGGACGCTT
AGCTAGGGG n(X3 to 10) CCCCTAGCT
AAGCGTCCT n(X3 to 10) CCCCTAGCT
AAGCGTCCT n(X3 to 10) AGGACGCTT
AGCTAGGGG n(X3 to 10) CCCCTAGCT

This resulted in the generation of 56 possible ZFN target sequences with mismatches and allowed spacing. The analysis identified only one sequence: AGCTAGGGGTTCTGGAGGACGCTT located in chromosome 8 within the *NF-YA8* coding sequence (exon 6). The custom shell script, ZFNsearch.sh, searched the specific patterns within the tomato genome. This tomato genome was previously prepared by the Prepare_Genome.sh script. This pipeline can ultimately generate files containing identified sequences along with 100x flanking sequence regions. This meticulous approach, combining targeted ZFN design and comprehensive off-target analysis, allowed for the identification and characterization of potential off-target sites, ensuring the safety and efficacy of ZFN-mediated genome editing in tomatoes.

Detailed bionformatic analysis

1. Method description

A Genome-wide **ZFN-site** tag scanner (ZFN-Site tool; https://epd.expasy.org/tagger/targetsearch.html) was initially used to identify possible off-targets. Specific parameters used, allowed for spacing of 5-6 nucleotides between tags, and a maximum of 2 sequence mismatches (2 per ZFN half-site). The 7 possible ZFN half-site pairs that were generated are as follows.

AGCTAGGGG AGGACGCTT
AAGCGTCCT CCCCTAGCT
AAGCGTCCT AGGACGCTT
AGCTAGGGG CCCCTAGCT
AAGCGTCCT CCCCTAGCT
AAGCGTCCT AGGACGCTT
AGCTAGGGG CCCCTAGCT

To further expand the search for any potential off-target effects of ZFN genome editing in tomatoes, a custom script named ZFNsearch.sh was developed utilizing the flexibility of Unix scripting. This script can be found in Github (DOI: 10.5281/zenodo.11369976) and enabled us to explore a wider range of parameters, including:

- Scanning the entire tomato genome.

- Filtering based on ZFN target recognition, while including variability in the distance between the two ZFN half sites (3 to 10 base pairs), as well as mismatch tolerance.

-Integrating flanking sequences around the ZFN half sites to leverage existing bioinformatics pipelines for efficient processing of potential hits (Blastn algorithm) [43].

This robust, in-house script empowered us to confidently interpret experimental results and ensure the integrity of ZFN-mediated genome editing by providing comprehensive off-target analysis.

The Genome assembly SL3.1 (RefSeq: GCF_000188115.5) was used as the reference genome. The only sequence identified was AGCTAGGGGTTCTGGAGGACGCTT. This sequence was subjected into additional blast for local alignment, including 100 bp upstream and downstream flanking sequences.

The final sequence was used to execute a Blastn analysis.

2. ZFNsearch Scrips

ZFNsearch.sh is a unix script that allows searches of any specific genomic sequence pair, interrupted by a given number of random nucleic acids. The search is possible for any given nucleotide FASTA sequence of interest or even whole genomes of any organism, given they are prepared by the also included unix script Prapare_Genome.sh.

This scrip can be applied for any pair of specific sequences that is interrupted by a specific number of random nucleotides, however it has been successfully applied to detect and report ZFN recognized targets, important for genome editing. The detected patterns will be reported in the command line, as well as in an output text file, that includes a sequence list of the detected patterns along flanking sequences (x100 bp on each side), a feature that enables further sequence analysis like Blast.

Steps of analysis (All files need to be in the same folder, or whole paths need to be provided)

1. Prepare nucleic sequence reference or whole genome (FASTA file) to search for patters (Need to do it first, and only once per sequence reference). In case you have used this sequence before go directly to step 2. Usage: bash Prepare_Genome.sh Any_FASTA_file
2. Search for patters Usage: bash ZFNsearch.sh Prep_Any_FASTA_file Pattern1 num_of_random_na Pattern2 Where Pattern1 = sequence num_of_random_na = number Pattern2 = sequence

The script will report the detected patterns in the terminal and output a txt file with a list of sequences found, including flanking sequences (x100 bp on each side).

3. Commands used

A variety of sequence patterns and random separated from 3 or 10 random nucleic acids were selected to execute the ZFNsearch.sh bash script. All costume scripts used can be found in GitHub (https://github.com/ikaramichali/ZFNsearch; DOI: 10.5281/zenodo.11369976).

The reference genome was downloaded and prepared as so:

wget https://ftp.ncbi.nlm.nih.gov/genomes/all/GCA/000/188/115/GCA_000188115.5_SL4.0/GCA_000188115.5_SL4.0_genomic.fna.gz

gunzip GCA_000188115.5_SL4.0_genomic.fna.gz

bash Prepare_Genome.sh GCA_000188115.5_SL4.0_genomic.fna

The starting ZFN-sites were designed using ZFN-Site tool (https://epd.expasy.org/tagger/targetsearch.html). Seven possible sites were proposed based on all available genome references. The command to search for the proposed ZFN sites, including intermediate nucleotides between the number 3 to 10, was prepared as so:

```
for i in {3..10}; do echo "\"AGCTAGGGG "$i" AGGACGCTT\" \"AAGCGTCCT "$i"
CCCCTAGCT\" \"AAGCGTCCT "$i" AGGACGCTT\" \"AGCTAGGGG "$i" CCCCTAGCT\"
\"AAGCGTCCT "$i" CCCCTAGCT\" \"AAGCGTCCT "$i" AGGACGCTT\" \"AGCTAGGGG
"$i" CCCCTAGCT\""; done
```

```
for i in "AGCTAGGGG 3 AGGACGCTT" "AAGCGTCCT 3 CCCCTAGCT" "AAGCGTCCT 3
AGGACGCTT" "AGCTAGGGG 3 CCCCTAGCT" "AAGCGTCCT 3 CCCCTAGCT"
"AAGCGTCCT 3 AGGACGCTT" "AGCTAGGGG 3 CCCCTAGCT" "AGCTAGGGG 4
AGGACGCTT" "AAGCGTCCT 4 CCCCTAGCT" "AAGCGTCCT 4 AGGACGCTT"
"AGCTAGGGG 4 CCCCTAGCT" "AAGCGTCCT 4 CCCCTAGCT" "AAGCGTCCT 4
AGGACGCTT" "AGCTAGGGG 4 CCCCTAGCT" "AGCTAGGGG 5 AGGACGCTT"
"AAGCGTCCT 5 CCCCTAGCT" "AAGCGTCCT 5 AGGACGCTT" "AGCTAGGGG 5
CCCCTAGCT" "AAGCGTCCT 5 CCCCTAGCT" "AAGCGTCCT 5 AGGACGCTT"
"AGCTAGGGG 5 CCCCTAGCT" "AGCTAGGGG 6 AGGACGCTT" "AAGCGTCCT 6
CCCCTAGCT" "AAGCGTCCT 6 AGGACGCTT" "AGCTAGGGG 6 CCCCTAGCT"
"AAGCGTCCT 6 CCCCTAGCT" "AAGCGTCCT 6 AGGACGCTT" "AGCTAGGGG 6
CCCCTAGCT" "AGCTAGGGG 7 AGGACGCTT" "AAGCGTCCT 7 CCCCTAGCT"
"AAGCGTCCT 7 AGGACGCTT" "AGCTAGGGG 7 CCCCTAGCT" "AAGCGTCCT 7
CCCCTAGCT" "AAGCGTCCT 7 AGGACGCTT" "AGCTAGGGG 7 CCCCTAGCT"
"AGCTAGGGG 8 AGGACGCTT" "AAGCGTCCT 8 CCCCTAGCT" "AAGCGTCCT 8
AGGACGCTT" "AGCTAGGGG 8 CCCCTAGCT" "AAGCGTCCT 8 CCCCTAGCT"
"AAGCGTCCT 8 AGGACGCTT" "AGCTAGGGG 8 CCCCTAGCT" "AGCTAGGGG 9
AGGACGCTT" "AAGCGTCCT 9 CCCCTAGCT" "AAGCGTCCT 9 AGGACGCTT"
"AGCTAGGGG 9 CCCCTAGCT" "AAGCGTCCT 9 CCCCTAGCT" "AAGCGTCCT 9
AGGACGCTT" "AGCTAGGGG 9 CCCCTAGCT" "AGCTAGGGG 10 AGGACGCTT"
"AAGCGTCCT 10 CCCCTAGCT" "AAGCGTCCT 10 AGGACGCTT" "AGCTAGGGG 10
CCCCTAGCT" "AAGCGTCCT 10 CCCCTAGCT" "AAGCGTCCT 10 AGGACGCTT"
"AGCTAGGGG 10 CCCCTAGCT"; do for p in $i; do bash ZFNsearch.sh
Prep_GCF_000188115.5_SL3.1_genomic.fna $p; done; done
```

Finally, the command

```
bash ZFNsearch.sh Prep_GCF_000188115.5_SL3.1_genomic.fna AGCTAGGGG 6 AGGACGCTT
> seqs_AGCTAGGGG_5_AGGACGCTT.fna
```

was the one that returned the only sequence match identified. The identified ZFN-site sequence was AGCTAGGGGTTCTGGAGGACGCTT.

The output including the 100x flanking sequence regions reaching the total size of 224 nucleotides, was as follows:

> seqs_6_Prep_GCA_000188115.4_SL3.1

AATACTCTTGCACTTTCCAACAATGAAATTGTGGTCTTATCCCATCTTTTGTATGACAGCC
ATATCTTCACGAGTCTCGACATCGTCATGCAATGAAGAG**AGCTAGGGGTTCTGGAGGA**
CGCTTTTTGAACACAAAGAATATGCAGGAATCCAAGCCTTCATCTCCAATGCACGACAG
AAATATCTTTAAGCGACAGGCAGGTGGCAACTTATCTAGTTCCATG

4. Sequence analysis

The final sequence was furthermore analyzed using Blastn [43]. Figure 2X shows the top sequence hits. All of them included the ZFN site found, varying in flanging sites. In tomato only one genomic site was found (AP009518.1), located in chromosome 8. The ZNF-site did exhibited variation between the sequences found, as shown in Figure 2X. The other tomato sequences were mRNA sequences of mostly predicted nuclear transcription factor Y subunit A-8 (LOC101246742). The tomato specific hits are described in **Table** S1.

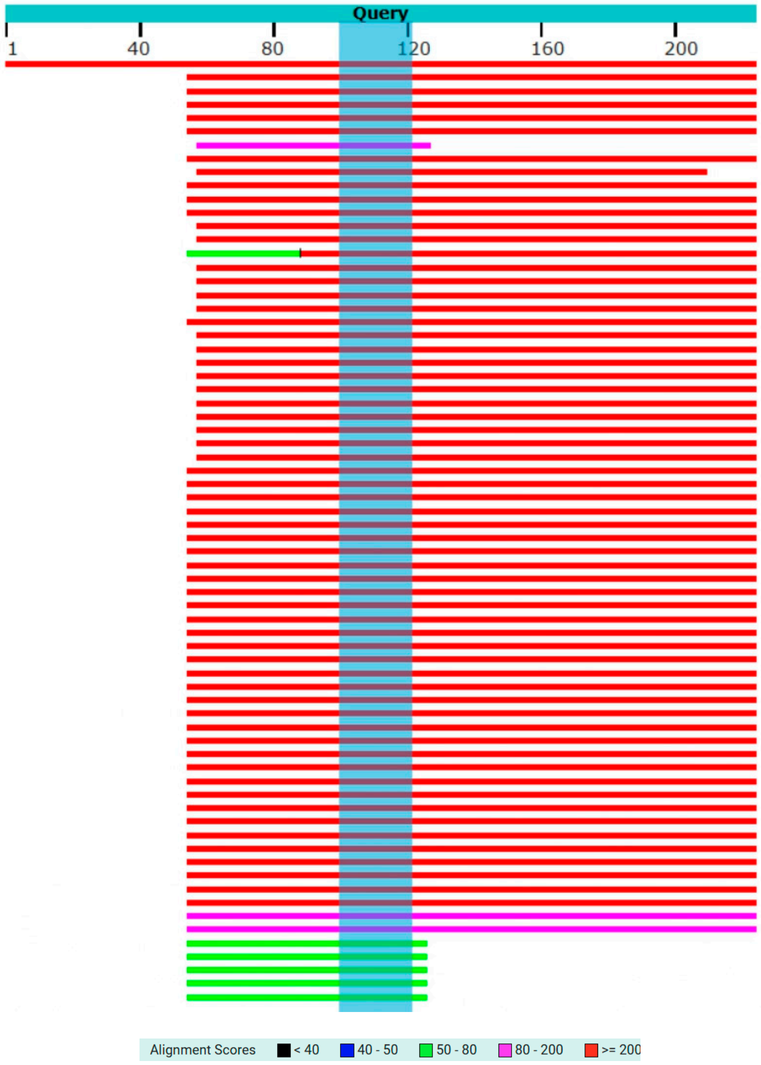

Figure S1A. Graphic summary of 70 top sequence hits of the identified sequence. The color of the lines indicates the identity score of the sequence in comparison to the query. The blue shadow indicates the ZFN-site location. The image was generated using Blastn

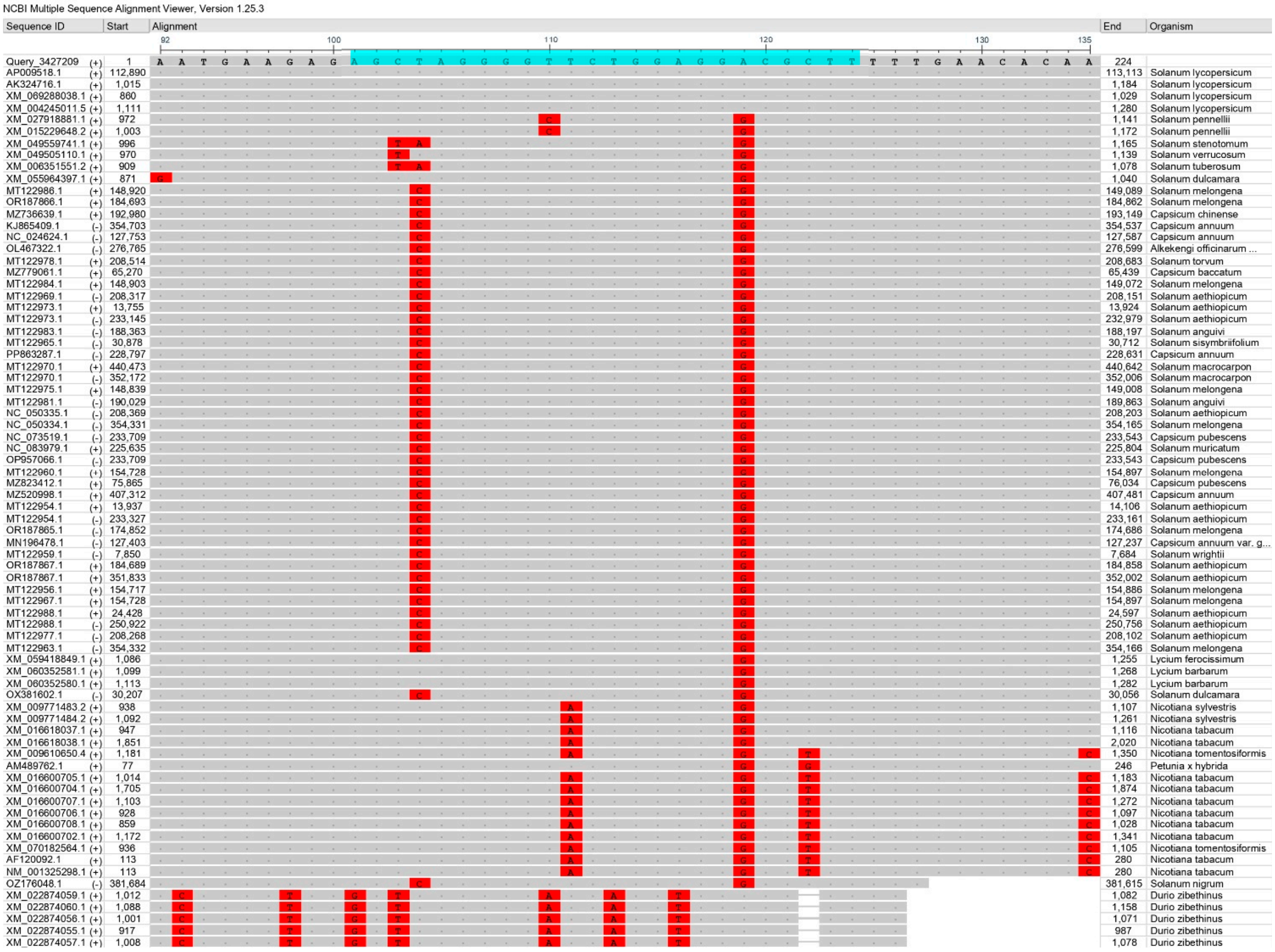

Figure S1B. Close sequence comparison between the best sequence hits found. Some sequence variation was detected within the ZNF-site (light blue), indicated by the color red. The image was generated using Blastn.and the tool MSA Viewer.

Table S1. Tomato specific sequence hits

Description	Scientific Name	Max Score	Total Score	Query Cover	E value	Per. ident	Acc. Len	Accession
Solanum lycopersicum DNA, chromosome 8, clone: C08HBa0067I16,	Solanum lycopersicum	414	414	100%	2e-111	100	127623	AP009518.1

complete sequence								
Solanum lycopersicum cDNA, clone: LEFL1081CB08, HTC in leaf	Solanum lycopersicum	309	309	76%	1e-79	99.41	1794	AK324716.1
PREDICTED: Solanum lycopersicum nuclear transcription factor Y subunit A-8 (LOC101246742), transcript variant X2, mRNA	Solanum lycopersicum	309	309	76%	1e-79	99.41	1652	XM_069288038.1
PREDICTED: Solanum lycopersicum nuclear transcription factor Y subunit A-8 (LOC101246742), transcript variant X1, mRNA	Solanum lycopersicum	309	309	76%	1e-79	99.41	1903	XM_004245011.5
